# Supplementary material for: Solvent Effects on Gelation Behavior of the Organogelator Based on L-Phenylalanine Dihydrazide Derivatives
Source: Materials (Basel). 2019 Jun 12;12(12):1890. doi: 10.3390/ma12121890 (PMC6631004; doi:10.3390/ma12121890)
Supplement: Supplementary file 1 [file materials-12-01890-s001.pdf]

Supporting Information

Solvent Effects on Gelation Behavior of the Organogelator Based on L-Phenylalanine Dihydrazide Derivatives

Yang Yu [1,*], Ning Chu [1], Qiaode Pan [1], Miaomiao Zhou [1], Sheng Qiao [1], Yanan Zhao [1], Chuansheng Wang [1] and Xiangyun Li [2,*,†]

[1] The Key Laboratory of the Inorganic Molecule-Based Chemistry of Liaoning Province and Laboratory of Coordination Chemistry, Shenyang University of Chemical Technology, Shenyang 110142, China; chuning0202@163.com (N.C.); pangqiaode89@163.com (Q.P.); dzjzzhoumiao@163.com (M.Z.); 18640104797@163.com (S.Q.); yanan1116@163.com (Y.Z.); wchsh18@163.com (C.W.)

[2] Yingkou Baoshan Ecology Coating Co., Ltd., Yingkou 115004, China

* Correspondence: yy_email@163.com (Y.Y.); lixiangyun@baoshangroup.com (X.L.)

† This author's contribution to the paper is the same as that of the first author.

Received: 30 March 2019; Accepted: 4 June 2019; Published: date

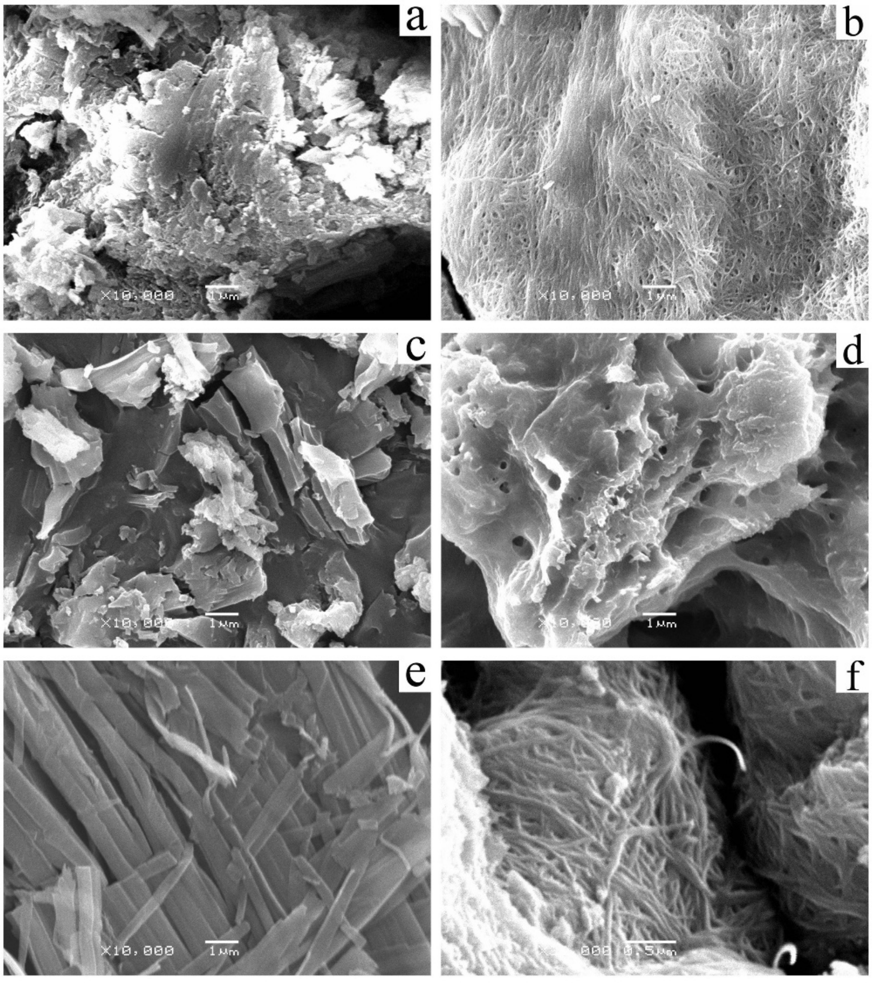

Figure S1. SEM images of xerogels formed by compound 4 (a) in methyl alcohol (2.0 wt%); (b) in n-propyl alcohol (4.0 wt%), (c) in n-butanol, (d) in iso-butanol, (e) in 1-pentanol and (f) in 1-hexanol.

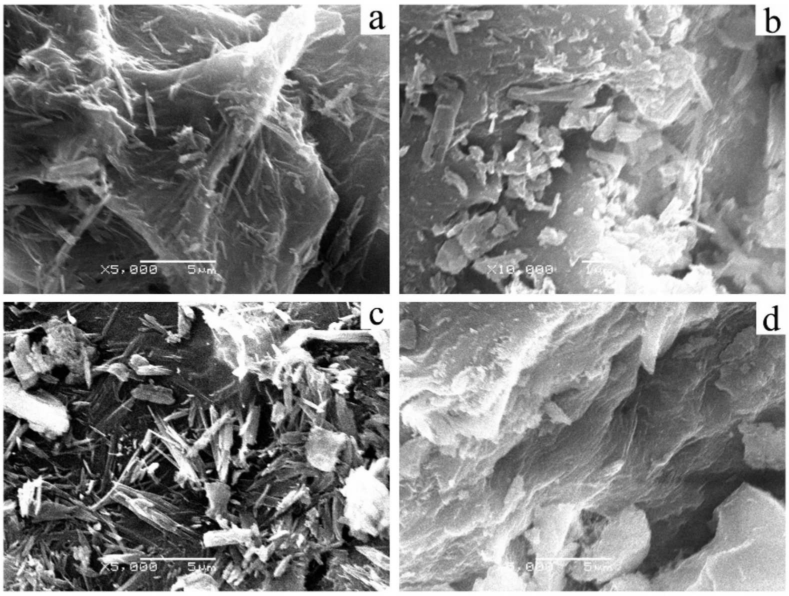

Figure S2. SEM images of xerogels formed by compound 4 (a) in n-propyl acetate (2.0 wt%), (b) in n-butyl acetate, (c) in isobutyl acetate and (d) in amyl acetate.
